# A case report of unusual presentation of a rare renal tumor

**DOI:** 10.1016/j.radcr.2023.03.042

**Published:** 2023-04-13

**Authors:** Farzaneh Sharifiaghdas, Behzad Narouie, Mohammad Ghasemi-Rad, Fatemeh Moosavian, Mohadese Ahmadzade, Hamidreza Rouientan

**Affiliations:** a Department of Urology, Urology and Nephrology Research Center, Shahid Labbafinejad Medical Center, Shahid Beheshti University of Medical Sciences, Tehran, Iran; b Department of Urology, Zahedan University of Medical Sciences, Zahedan, Iran; c Department of Interventional Radiology, Baylor College of Medicine, 1 Baylor Plaza, Houston, TX 77030, USA; d Department of Radiology, Shahid Beheshti University of Medical Sciences, Tehran, Iran

**Keywords:** Renal Ewing sarcoma, Primary renal ESFT, Chemotherapy

## Abstract

There are very few cases of primary renal Ewing sarcomas, which are characterized by a high rate of metastasis. These tumors are often mistaken for other more common kidney tumors due to their rarity and lack of pathognomonic symptoms in the early stages. A 28-year-old male patient presented to our clinic with a 2-month history of nonproductive progressive cough and left flank pain. The chest was scanned with contrast-enhanced computed tomography, which showed a heterogeneously enhancing mass with central vascularity on the left retroperitoneal. An abdominal dynamic multiphasic magnetic resonance imaging with contrast revealed a large mass that was highly suggestive of neoplastic pathology and multiple metastatic nodules. The pathology report indicated a renal Ewing sarcoma as the result of a core needle biopsy. In order to initiate chemotherapy promptly, early detection is crucial, and radiology plays an important role in diagnosing.

## Introduction

The Ewing sarcoma family of tumors (ESFT) represents a group of the previous terminologies such as Ewing sarcoma of bone, extra-osseous Ewing sarcoma, peripheral primitive neuroectodermal tumor, Askin tumor of thoracopulmunary region that are characterized by small round blue cells on histopathology and show similar chromosomal translocations [1].

Primary renal ESFTs is clinically rare, accounting for less than 1% of renal tumors and have been characterized by a high potential for rapid metastasis [2,3].

Due to their rarity and not having pathognomonic symptoms in early stages, these tumors are easily mistaken for other more common tumors arising from the kidney. The 4-year overall survival for patients without metastasis is 85% compared to 47% for patients with metastasis [4]. In addition, there is no consensus on the optimal management of these tumors due to the paucity of available data.

We report the case of a young male with primary renal ESFTs that presented to us with an unusual symptom.

## Case presentation

A 28-year-old male patient referred to our clinic with a 2-month history of nonproductive progressive cough and left flank pain. After undergoing a contrast-enhanced computed tomography scan of his chest to determine the cause of his cough, a heterogeneously enhancing mass with central vascularity was partially visualized on the left retroperitoneum. On presentation, his vital signs were stable and apart from abdominal distention with a large mass in the left upper quadrant and left flank tenderness, his physical examination was otherwise unremarkable.

**Fig. 1:**
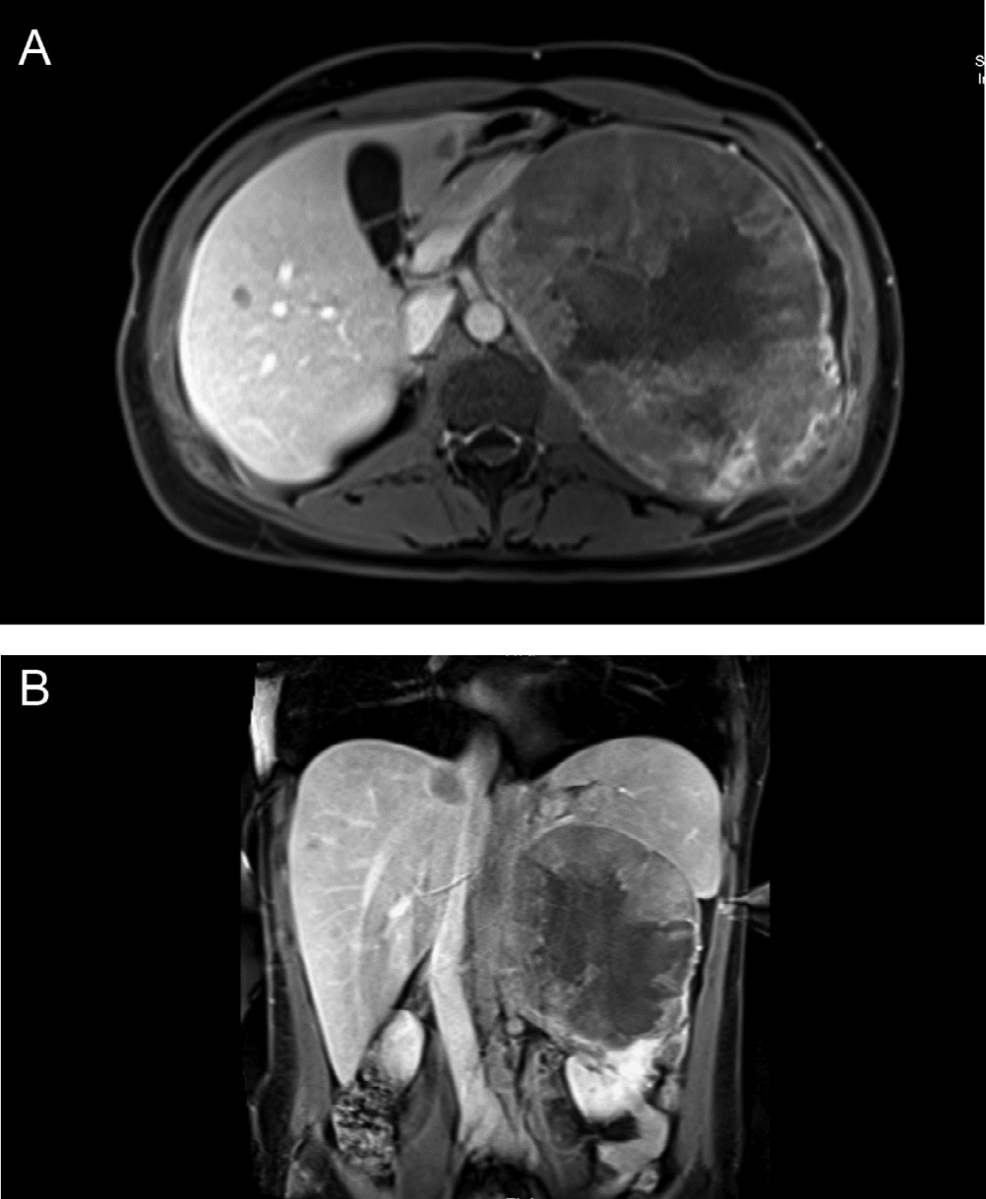
Axial (A) and coronal (B) postcontrast magnetic resonance imaging of the abdomen demonstrated a large heterogeneously enhancing mass with central necrosis arising from the left kidney.

For further evaluation, dynamic multiphasic magnetic resonance imaging of the abdomen with contrast showed a large mass measuring 155 × 140 × 150 mm with fine lobulated margin and central cystic changes in left suprarenal fossa with diffusion restriction and heterogeneous pattern of enhancement with a metastatic lymph node at left side of abdominal aorta and multiple metastatic nodules in the lower lobes of both lungs and liver which was highly suggestive of neoplastic pathology (Fig. 1). Also, total body bone scan performed for staging revealed no bone metastasis.

**Fig. 2:**
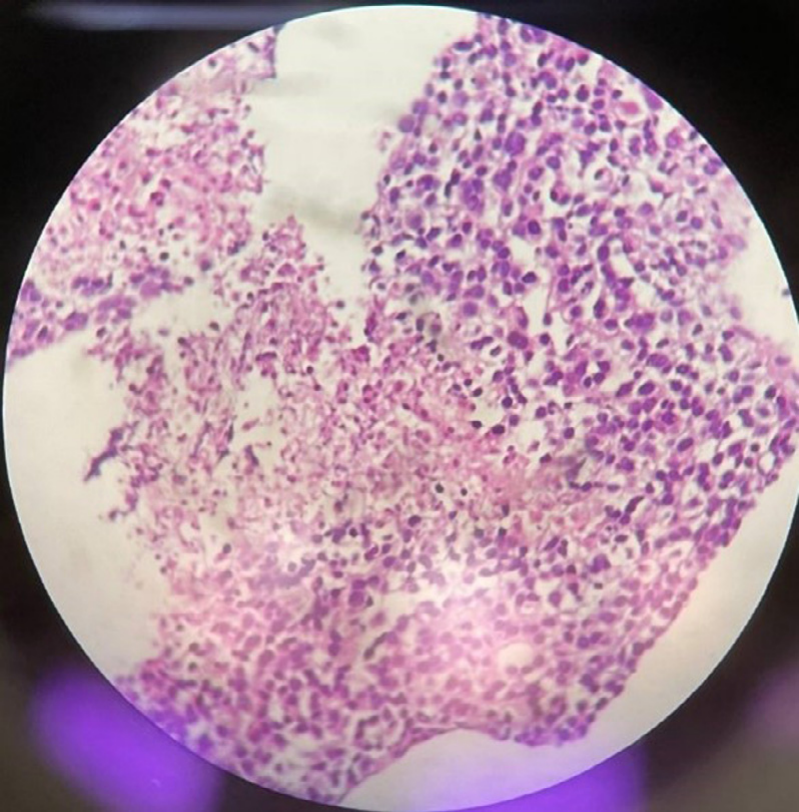
Tiny fragments of malignant small cell tumors with necrosis. (Hematoxylin and eosin-stained section (40×) shows tumor cells arranged in sheets with hyperchromatic nuclei and scant cytoplasm.)

After multidisciplinary consult including urologist, oncologist, and radiologist, the patient underwent ultrasound-guided core needle biopsy of the renal mass. Microscopic examination of the hematoxylin and eosin-stained sample showed small round cells (Fig. 2).

On immunohistochemistry (IHC), the tumor cells were negative for LCA, WT-1, DESMIN, Myogenin, phox-B2, CD99, SOX10, Chromogranin, and GATA3 and positive for CK, FLI-1, and Synaptophysin. The pathology report was compatible with renal PNET/Ewing sarcoma.

This patient was diagnosed as metastatic (stage 4) upon presentation and was being treated with a combination of chemotherapy regimen including vincristine, doxorubicin, and cyclophosphamide.

## Discussion

Approximately 60% of cases of primary renal ESFT occur in young males, with an average age of 30.4 years at the time of diagnosis [5]. The majority of patients present with nonspecific symptoms, including hematuria, pain, and palpable abdominal masses. Nevertheless, the main complaint of our patient was a persistent, nonproductive cough.

In view of its atypical location, renal ESFT is diagnosed through an integrated approach encompassing histomorphology, IHC, and confirmatory molecular genetic testing. The light microscope demonstrates small round blue cells with a high nucleocytoplasmic ratio that can be arranged in sheets and form Homer-Wright rosettes. Tumor cells exhibit neuro-ectodermal IHC markers, including neuron-specific enolase and synaptophysin.

Nephrectomy in conjunction with neoadjuvant and adjuvant chemotherapy has become the accepted standard of treatment for nonmetastatic renal ESFT [4]. In patients with metastatic disease, chemotherapy alone may be sufficient for long-term survival, resulting in complete remission and possibly cure in a few patients [6]. The most commonly used chemotherapy regimen, according to ESFT summary and treatment policy, is vincristine, doxorubicin (or Adriamycin), and cyclophosphamide. At least 9 weeks before surgery or radiation, 2 combinations of drugs are given every 2 or 3 weeks, and then more chemo is given afterward. A total of 14-15 cycles of chemotherapy are usually given [4,7]. Based on a study by Teegavarapu et al. [8], neoadjuvant chemotherapy is rarely administered to patients with ESFT, often due to early total tumor resection without diagnostic biopsy. Clinical experiences at several sarcoma specialist units have dispelled concerns regarding disease local recurrence in the biopsy needle tract; a study of 358 patients undergoing percutaneous biopsy of retroperitoneal sarcoma found only 1 case of suspected needle tract seeding over a median follow-up period of 44 months (0.5%) [9].

Our case report emphasizes the importance of performing a diagnostic biopsy prior to nephrectomy in patients with atypical renal masses, particularly those with metastatic disease. An important objective is to avoid unnecessary surgery since a nephrectomy could delay or even prevent the delivery of effective systemic chemotherapy, such as high-dose ifosfamide. Additionally, recently approved antitumor agents, such as apatinib, were promoted as potential therapeutic options for patients suffering from ESFTs [10].

Even though neoadjuvant therapy, surgery, and adjuvant therapy have improved the survival rate of patients with renal ESFT, future research is needed to validate their recurrence-free and overall survival.

In the presence of a renal mass, especially if it is large, it is imperative to keep in mind that primary renal ESFT may occur primarily in the kidney. Ultimately, a diagnosis is determined by histopathology with IHC. An early diagnosis is essential for prompt treatment initiation, and radiology plays an increasingly important role in the diagnosis, staging, monitoring, and surveillance of treatment.

## Patient consent

A written informed consent for publication of this case was obtained from the patient.
